# Integrating chromosomal aberrations and gene expression profiles to dissect rectal tumorigenesis

**DOI:** 10.1186/1471-2407-8-314

**Published:** 2008-10-29

**Authors:** Esther H Lips, Ronald van Eijk, Eelco JR de Graaf, Jan Oosting, Noel FCC de Miranda, Tom Karsten, Cornelis J van de Velde, Paul HC Eilers, Rob AEM Tollenaar, Tom van Wezel, Hans Morreau

**Affiliations:** 1 Department of Pathology, Leiden University Medical Center, PO Box 9600, 2300 RC Leiden, the Netherlands; 2 Department of Surgery, Leiden University Medical Center, PO Box 9600, 2300 RC Leiden, the Netherlands; 3 Department of Medical Statistics, Leiden University Medical Center, PO Box 9600, 2300 RC Leiden, the Netherlands; 4 Department of Surgery, IJsseland Hospital, P.O. Box 690, 2900 AR Capelle a/d Ijssel, the Netherlands; 5 Department of Surgery, Reinier de Graaf Gasthuis, P.O. Box 5011, 2600 GA Delft, the Netherlands

## Abstract

**Background:**

Accurate staging of rectal tumors is essential for making the correct treatment choice. In a previous study, we found that loss of 17p, 18q and gain of 8q, 13q and 20q could distinguish adenoma from carcinoma tissue and that gain of 1q was related to lymph node metastasis. In order to find markers for tumor staging, we searched for candidate genes on these specific chromosomes.

**Methods:**

We performed gene expression microarray analysis on 79 rectal tumors and integrated these data with genomic data from the same sample series. We performed supervised analysis to find candidate genes on affected chromosomes and validated the results with qRT-PCR and immunohistochemistry.

**Results:**

Integration of gene expression and chromosomal instability data revealed similarity between these two data types. Supervised analysis identified up-regulation of *EFNA1 *in cases with 1q gain, and *EFNA1 *expression was correlated with the expression of a target gene (*VEGF*). The *BOP1 *gene, involved in ribosome biogenesis and related to chromosomal instability, was over-expressed in cases with 8q gain. *SMAD2 *was the most down-regulated gene on 18q, and on 20q, *STMN3 *and *TGIF2 *were highly up-regulated. Immunohistochemistry for SMAD4 correlated with *SMAD2 *gene expression and 18q loss.

**Conclusion:**

On basis of integrative analysis this study identified one well known CRC gene (*SMAD2*) and several other genes (*EFNA1, BOP1, TGIF2 *and *STMN3*) that possibly could be used for rectal cancer characterization.

## Background

Accurate staging of rectal tumors is essential for choosing the correct treatment. Small pedunculated adenomas can be removed by snare excision, while large sessile adenomas can be cured by transanal endoscopic microsurgery [1]. For carcinomas, total mesorectal excision with preoperative radiotherapy is the gold standard [2]. However, preoperative staging using histology and modern imaging techniques is not always adequate, resulting in either under- or over-treatment. Therefore in current practice, additional markers indicating the aggressiveness of the tumor to be resected are extensively investigated [3,4]. It is of utmost importance to have parameters that can discriminate large benign adenomas from adenomas with a small invasive focus, as well as carcinomas with and without lymph node metastasis.

Recently, studies have investigated the application of microarrays in the diagnosis and prognosis of various stages of colorectal cancer (CRC). Gene expression signatures have been published that discriminate adenomas from carcinomas, Dukes B and C CRC, as well as lymph node positive and negative CRC patients [5-8]. Other studies using array comparative genomic hybridization (aCGH) describe specific genomic alterations related to different stages of colorectal cancer [9-12]. While there is little overlap between gene lists obtained from expression studies, common genomic alterations involved in CRC progression are established [13,14]. Chromosome 8q, 13q, and 20 gain occur early in the establishment of primary CRCs, loss of 4p is associated with the transition from Dukes' A to B-D. Deletion of 8p and gains of 7p and 17q are correlated with the transition from primary tumor to liver metastasis, whereas losses of 14q and gains of 1q, 11, 12p, and 19 are late events (reviewed in [14]).

Several studies have previously integrated gene expression profiles and genomic alterations in CRC and found a good correlation between both data types [15-19]. Tsafrir et al. [19] found that often, large chromosomal segments, containing multiple genes, are transcriptionally affected in a coordinated way, and suggested that the underlying mechanism is a corresponding change in DNA content. Furthermore, they showed that these aberrations are absent in normal colon mucosa, appear in benign adenomas, become more frequent as disease advances, and are found in the majority of metastatic samples. In contrast, Platzer et al. found that underexpression was more common than overexpression in amplified regions[20], and a study by Staub et al. found that deleted regions usually show underexpression while amplified regions exhibit heterogenous expression. For several gene islands of deregulated expression chromosomal aberrations have never been observed [18].

While those studies mainly analyzed how chromosomal aneuploidies affect global gene expression, we used an integrative approach to identify specific candidate genes for staging rectal tumors. In a previous study, we showed that loss of 17p, 18q12-22 and gain of 8q22-24,13q and 20q could accurately distinguish adenoma from carcinoma tissue, and that gain of 1q23 was correlated to lymph node metastasis [21]. In the present study, we identify target genes on the affected chromosomes and validate the microarray data by means of quantitative RT-PCR and immunohistochemistry. We believe that this integrative approach generates more accurate and robust data than either data type alone.

## Methods

### Samples

Sixty-six fresh-frozen operated tumor samples were derived from a previous study in which copy number aberrations were determined [21]. In addition, material from 13 other cases was obtained. The samples were from patients treated by TEM from the IJsselland Hospital and Reinier de Graaf Hospital, the Netherlands, or from the TME trial, a Dutch multicenter trial in which 1530 patients were included from 1996 to 1999 [2]. None of the patients received radiotherapy or other adjuvant therapy. All samples were reviewed by a pathologist (H.M.), dysplasia was scored, and tumor percentage was assessed (50–80%) in a frozen section of the tissue. Intramucosal carcinomas were considered as adenoma with high grade dysplasia, as opposed to invasive carcinoma [22]. The local medical ethical committee approved the study (protocol number P04.124).

### RNA isolation

Tumors were macrodissected in a cryostat by removing surrounding non-neoplastic tissue. Twenty 30-μm sections were cut from each tumor. To guide microdissection, a 4-μm section was cut and haematoxylin and eosin stained, before the first section, and after the tenth and twentieth section, and assessed for the presence of adenoma or carcinoma tissue, or a mixture of both. RNA was isolated with RNAzol reagent (Tel-Test Inc., Friendswood, TX) according to the manufacturer's protocol and was purified using the Qiagen RNeasy mini kit with on-column DNase digestion, according to manufacturer's instructions (Qiagen Sciences, Germantown, MD). The quality of the RNA was assessed with lab-on-a-chip using the Agilent 2100 Bioanalyzer (Agilent Technologies, Palo Alto, California).

### Microarray analysis

Two μg of total RNA was amplified and labeled using Ambion's Amino Allyl MessageAmp™ aRNA kit and protocol (Ambion Inc., Austin, TX). The quality of each aRNA was checked by lab-on-a-chip (Agilent Technologies). Dye incorporation was checked with a Nanodrop (Wilmington, DE). For each microarray experiment, 2.0-μg aliquots of aRNA were labeled with Cy5 (Amersham Biosciences, Buckinghamshire, UK). The labeled aRNAs were mixed with equal amounts of Cy3-labeled reference aRNA, consisting of pooled RNAs isolated from five colorectal cancer cell lines (HCT116, LS411N, SW480, HCT15, Caco2) and five normal rectum samples. To the mixture of labeled reference and sample RNA, 20 μg human COT-1 DNA (Invitrogen, Carlsbad, CA), 8 μg yeast tRNA (Invitrogen) and 20 μg polyadenylic acid (Sigma-Aldrich, St. Louis, MO) were added. Preheated hybridization buffer (25% formamide, 5 × SSC, 0.1% SDS) was added just before overnight hybridization at 42°C to human 35 K oligo microarrays, manufactured at the Central Microarray Facility (CMF) of the Netherlands Cancer Institute. Protocols, GeneID list and information about arrays are available at the website of the CMF . Hybridization slides were washed and scanned using the Agilent G2565BA Microarray Scanner (Agilent Technologies); spot intensities were extracted from the scanned images with Genepix 5.1 (Axon, Baden, Switzerland).

### Data analysis

Raw intensity data (.gpr files) were analyzed in the R environment . The Limma (linear models for microarray data) package of Bioconductor  was used for importing the data, normalizing the arrays and identifying differentially expressed genes. Control spots and spots with more than 10% saturation, a diameter smaller than 60 μm or signal intensity less than 20 counts above background were excluded from the analysis. Data were corrected for local background (method normexp) and normalized within arrays by print-tip loess normalization and between arrays by quantile normalization. Duplicate experiments were performed for eight different tumor samples, showing Pearson correlation coefficients ranging from 0.92 to 0.97.

Statistically significant differences in gene expression were assessed using a moderate empirical Bayes test statistic available through Limma. The B-value is the log-odds that a gene is differentially expressed. The obtained *p*-values were controlled for false discovery using the Benjamini and Hochberg procedure. Oligos with corrected *p*-values ≤ 0.001 were considered statistically significant.

In the integrated analysis, the gene expression levels were normalized per gene by subtracting the average gene expression of a reference sample set consisting of the adenomas with a limited amount of genomic changes (maximum of two small aberrations). Chromosomal plots of expression values were made in R by smoothing and integrated analysis [21,23]. Heat maps of expression data of specific chromosomes were generated in Spotfire DecisionSite (Spotfire, Sommerville, MA). For supervised analysis, we used Statistical Analyses of Microarrays (SAM) [24]. We analyzed every affected chromosome arm separately in SAM to find specific genes related to that specific chromosomal alteration. Groups were made on the basis of loss or gain and retention of a specific chromosome, determined by SNP array analysis [21]. For the analysis of gene expression levels of individual samples of newly identified and well-known colorectal cancer genes t-tests were performed using SPSS 12.0 (SPSS Inc, Chicago, IL, USA).

The data discussed in this publication have been deposited in NCBIs Gene Expression Omnibus (GEO) . The genomic data from the SNP arrays are accessible through GEO Series accession number GSE7946, while the gene expression array data are accessible through GEO Series accession number GSE12225.

### Quantitative RT-PCR (qPCR)

Two micrograms of total RNA was reverse-transcribed with AMV Reverse Transcriptase (Roche, Penzberg, Germany). Real-time reverse transcriptase (RT) PCR was carried out in an 7900 HT Real Time PCR System (PE Applied Biosystems, Foster City, CA) in a 10 μl volume containing 1× qPCR SYBR Green/ROX PCR Mastermix (SuperArray, Frederick, MD) and 1 μl RT^2 ^primer set using the following PCR profile: 10 minutes at 95°C, followed by 40 cycles of 15 seconds at 95°C and 1 minute at 60°C. Primers used for real-time RT-PCR were targeted against *SMAD2, VEGF, EFNA1, BOP1, and STMN3*. Primer sequences for the target gene *SMAD2 *were 5'-ATTTGCTGCTCTTCTGGCTCAG-3' and 5'-ACTTGTTACCGTCTGCCTTCG-3' and for *VEGF *5'-AAACCCTGAGGGAGGCTCC-3' and 5'-TACTTGCAGATGTGACAAGCCG-3'; for *EFNA1*, *BOP1*, and *STMN3*, we used RT^2 ^PCR Primer sets (SuperArray, Frederick, MD). Candidate genes for normalization were selected on the basis of showing the least variation between all samples (*CPSF6, GAPDH *and *EEF1A*). In all 79 samples the expression of these three genes was measured. Normalization was based on geometric averaging of the candidate normalization genes, as previously described [25], to acquire a reliable normalization of the qPCR experiments. This method provides a normalization factor (NF), representative of the amount of mRNA in each sample. Subsequently the expression of the gene of interest was divided by this normalization factor. The obtained normalized expression data were log2 transformed and analyzed in SPSS 12.0 (SPSS Inc, Chicago, IL, USA).

### Immunohistochemistry

BOP1 staining was performed on 4 μm thick fresh frozen tumor sections, using standard procedures. SMAD4 staining was performed on tissue micro-arrays (TMA), as previously described [26]. For the formalin fixed paraffin embedded tissue antigen retrieval was performed by boiling the slides for 10 min in Tris-EDTA pH 8.0 (SMAD4) using a microwave oven, after which the sections were cooled in this buffer for at least 2 h at room temperature. TMA sections were then rinsed in demineralized water and phosphate buffered saline (PBS). The frozen tissue sections were incubated for one hour with a 1:100 dilution of BOP1 cell supernatant (Ascension, Munich, Germany) and the TMA sections with SMAD4 (clone B-8, sc-7966, Santa Cruz Biotechnology, Santa Cruz, CA; dilution 1:100). Sections were washed in PBS and incubated with biotinylated rabbit anti-rat (1:200; DAKO, Glostrup, Denmark) and streptavidin-biotin complex (1:100; DAKO) (BOP1) or Envision HRP-ChemMate kit (DAKO) (SMAD4) for 30 min. Diaminobenzidine tetrahydrochloride was used as a chromogen for BOP1 staining. All tumor specimens were stained simultaneously to avoid interassay variation. BOP1 staining was categorized as no expression (IHC-score 0), weak expression (1), moderate expression (2) and strong expression (3). SMAD4 was scored in the following categories: no nuclear staining with a positive internal control (total loss) (0), weak nuclear staining (down regulation) (1), and moderate to strong nuclear staining (positive) (2, 3). The mean expression of three punches per patient was assessed for SMAD4.

## Results and discussion

### Sample description

In a previous study, we built a rectal cancer progression model based on five "malignant" genomic alterations (loss of chromosomes 17p and 18q12-22 and gain of chromosomes 8q22-24, 13q, and 20q) [21]. In addition, gain of 1q23 was associated with lymph node metastasis. We assumed that integrating genomic and gene expression data would allow the identification of important genes for rectal tumor staging. Therefore, we obtained gene expression profiles from 66 samples, which were also typed for LOH and copy number abnormalities in the previous study [21]. From 13 additional samples, only gene expression measurements were available. Adenoma tissue was subdivided into pure adenomas (A/A) and adenoma fractions from cases with a carcinoma focus (A/C). The carcinoma tissue was subdivided into tumor fractions consisting of a mixture of adenoma and carcinoma tissue (AC/C), carcinomas without lymph node metastasis (C/C) and carcinomas with lymph node metastasis (C/C (N+)). Sample characteristics and genomic data are summarized in Table 1.

**Table 1:** Summary of clinical and pathological data of 79 tumor samples

	A/A	A/C	AC/C	C/C	C/C(N+)
Tissue fraction analyzed	Adenoma	Adenoma	Adenoma and carcinoma mixture	Carcinoma	Carcinoma

Tumor stage	Adenoma	Carcinoma	Carcinoma	Carcinoma	Carcinoma

Treatment					
TEM	24	6	5	3	1
TME	4	3	2	20	11

Sex (M/F)	15/13	2/7	3/4	10/13	10/2

Age (mean)	69	71	64	66	62

Dysplasia (adenoma)					
Low	15				
high	9				

Stage (carcinoma)					
T1		7	3	11	
T2		2	3	12	12
T3			1		

Size(cm) (mean)	5.9	4.4	4.2	3.2	5.1

Genomic data					
Samples typed(n)	21	8	7	19	11
Abberations (%)					
1q23 gain	0	9	11	14	62
8q22-24 gain	9	18	44	50	62
13q gain	4	36	67	59	85
17p loss	17	18	44	91	62
18q12-22 loss	17	36	56	86	77
20q gain	17	27	78	86	92

### Global analysis of gene expression and genomic data

**Figure 1:**
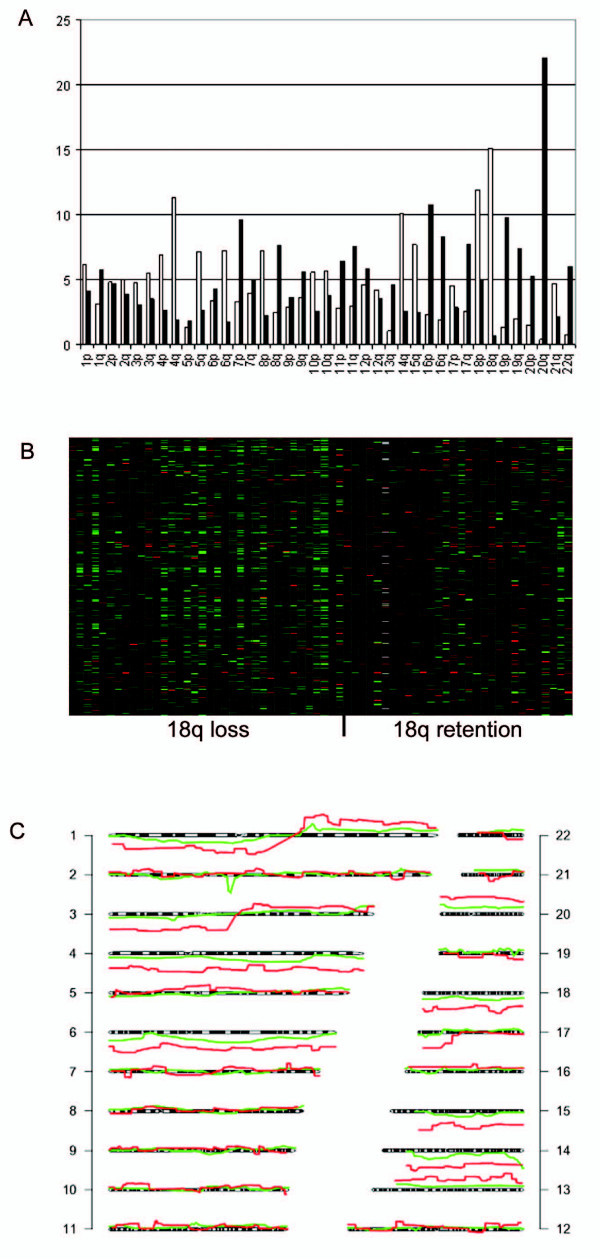
**Visual depiction of the similarity between gene expression and genomic data**. A. Summary of gene expression data for all 79 samples. The distribution of 2853 progression genes over the chromosome arms is shown. The x-axis shows all chromosome arms, the y-axis shows the percentage of genes for a certain chromosome arm that is differentially expressed. White bars represent downregulated genes, black bars represent upregulated genes. B. Heat map of all gene expression data for the 66 samples with 18q genomic data available. Every column represents a single sample. Samples on the left side show loss of 18q, while samples on the right side show retention of chromosome 18q, both measured by SNP arrays. The y-axis shows all 18q genes from the centromere to telomere. Red lines indicate genes with a higher expression, green lines a lower expression. C. Chromosomal plot of sample 203 (carcinoma with lymph node metastasis) based on SNP array data (red) and gene expression array data (green).

First, we performed a global analysis of the similarity between gene expression and genomic data as a verification of the data. We determined if gene expression changes between tumor stages were also differently regulated on the chromosomal level. Analysis of the chromosomal location of 2853 genes that showed a trend in expression over the subsequent tumor stages (A/A- A/C- AC/C- C/C- C/C(N+)) ("progression genes", Additional file 1) revealed that genes on chromosome 18q were most frequently down-regulated and genes on chromosome 20q were most frequently up-regulated (Figure 1A), which was expected based on our genomic data [21]. Heat maps of gene expression patterns for particular chromosomes were made. A representative heat map for all the genes on chromosomes 18q showed that samples with 18q loss had a lower gene expression than samples with 18q retention (Figure 1B). Also concordance in individual samples was seen: gene expression data was plotted along the chromosome and compared to the patterns obtained by the genomic arrays (Figure 1C). Although the patterns are not exactly similar, for many chromosomes a clear resemblance is observed.

As verification we also determined if gene expression levels of newly identified genes from the progression analysis and well-known colorectal cancer gene sets were also differently regulated on the chromosome level. We focused on the genes on chromosome 1q, 8q, 13q, 17p, 18q and 20q. We compared gene expression values for the samples with gain or loss of the respective chromosome versus the samples with retention. The progression genes on chromosomes 1, 8, 13, and 20 respectively, show a higher expression in the samples with gain, while genes on chromosome 18 show a lower expression in the samples with 18q loss (Additional files 1 and 2). Some of the colorectal cancer genes show a significant change in the same direction as the chromosomal change (e.g. *SMAD4, MUC1, BCL2*) while for others this is not evident (i.e. *p53, BMP7*). It is worth mentioning that for several of these well known colorectal cancer genes possible differential expression cannot be detected since their expression level is too low on the microarrays.

### Further identification of potential candidate genes in altered genomic regions

To identify specific genes of pathologic relevance in the affected chromosomal regions, we performed supervised analysis using the Significance Analysis of Microarrays package [24], with groups based on the specific chromosomal alterations. This analysis was done for the five "malignant" chromosomes and 1q. We examined whole chromosome arms as many patients had lost the whole arm. With a minimal fold change of 1.5 and a false discovery rate (FDR) <10%, we identified, respectively, 39, 30, 38, 20, 36, and 32 significant genes in relation to 1q gain, 8q gain, 13q gain, 17p loss, 18q loss and 20q gain (Additional file 1). All expression changes were in the expected direction, with the gain of 8q as an exception, showing not only 30 up-regulated genes, but also 3 genes that were down-regulated. The genes on chromosome 20q had the highest fold change.

We next focused on four genes identified in the supervised analyses. *EFNA1 *on 1q, *BOP1 *on 8q, *SMAD2 *on 18q and *STMN3 *on 20q, were selected, based on a high fold change and a low false discovery rate (Additional file 1). These genes were in the specific listed regions (*SMAD2 *at 18q21, *BOP1 *at 8q24), with an exception for 1q, as most tumors with gene expression values had lost chromosome 1q entirely. Moreover, these genes were previously shown to be involved in (colorectal) cancer [27-30]. qPCR and immunohistochemistry were used to validate their expression in individual cases.

### Validation by qPCR

**Figure 2:**
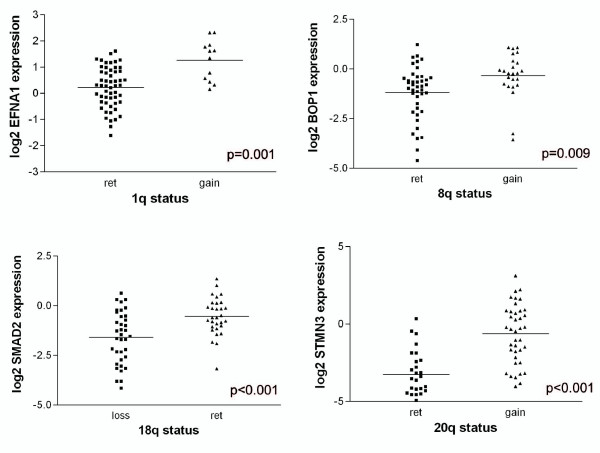
**Validation of array data by RT-PCR**. Plots of relative gene expression (log2 values) measured with RT-PCR are shown. The x-axis shows the samples, the y-axis shows the log2 relative gene expression value. Samples with retention are compared with samples with loss (18q) or gain (1q, 8q and 20q) of a specific chromosome arm. The line indicates the mean. P-values were computed by Student's t-test.

**Figure 3:**
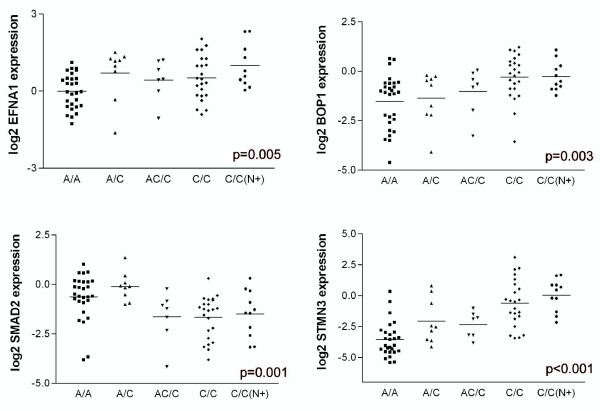
**Expression of *EFNA1, BOP1, SMAD2 and STMN3 *in the different patient groups**. Plots of relative gene expression (log2 values) measured with RT-PCR are shown. The x-axis shows the different sample groups. The y-axis shows the relative log2 gene expression data. Lines indicate the means. According to ANOVA analysis, the expression of all genes was significantly different between the groups.

To confirm the association between chromosomal aberrations and specific genes, we performed validation of expression data by qPCR. Correlation coefficients between expression array data and qPCR data were 0.71, 0.49, 0.81 and 0.91 (p < 0.001, for all four genes) for *EFNA1, BOP1, SMAD2 *and *STMN3*, respectively. Additionally the relation between specific genes and genomic regions was validated: *SMAD2 *was less expressed in the samples with 18q genomic loss (p < 0.001), while *EFNA1, BOP1 *and *STMN3 *were all more expressed in samples with gains of 1q, 8q and 20q, respectively (p = 0.001, p = 0.009 and p < 0.001) (Figure 2). When the different sample groups were compared for expression of these four genes, their pattern of expression accompanied the genomic alterations: *SMAD2 *was less expressed in the carcinomas, while *EFNA1, BOP1 and STMN3 *all showed an increased expression in the malignant tumor fractions; EFNA1 was also notably expressed in the A/C fractions (Figure 3). The chromosomal changes and accompanying gene expression changes show thus a good trend with the increasing malignancy of the tumor stages.

### 1q gain and EFNA1

**Figure 4:**
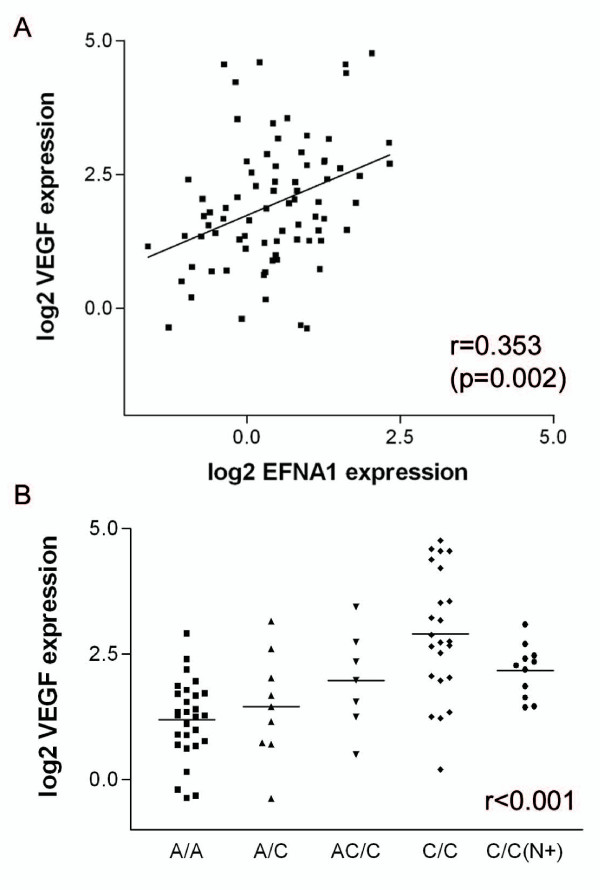
**Correlation of *EFNA1 *with *VEGF *expression values**. A. Correlation plot of relative log2 *EFNA1 *mRNA expression (x-axis) and relative log2 *VEGF *mRNA expression (y-axis). B. Relative log2 *VEGF *expression (y-axis) in the different clinical groups (x-axis).

Previously, we found that gain of 1q might be related to lymph node metastasis in rectal cancer [21]. Samples with gain of 1q showed a higher expression of two probes for *EFNA1 *than samples with 1q retention (Additional file 1 and Figure 2). *EFNA1 *is a ligand for Eph receptor tyrosine kinases and plays a key role in the migration and adhesion of cells during development [31]. Recently, it was found to be related to tumor-induced neovascularization [27]. Brantley-Sieders et al. found that *EFNA1 *over-expression elevated vascular endothelial growth factor (VEGF) levels, suggesting that *EFNA1*-mediated modulation of the VEGF pathway is a mechanism by which *EFNA1 *regulates angiogenesis [32]. VEGF plays a key role in angiogenesis during tumor growth and metastasis [33]. We measured *VEGF *mRNA expression by qPCR and found that it was correlated with *EFNA1 *mRNA expression (r = 0.353, p = 0.002) (Figure 4). *EFNA1 *and *VEGF *showed increased expression in the carcinomas in comparison to adenomas. This was expected, as neo-angiogenesis is an important factor in malignant transformation. However, in lymph node positive carcinomas *VEGF *expression appears to be down-regulated as compared to node negative rectal cancers whereas such a trend is not observed for *EFNA1*. An explanation for this observation does not seem obvious. As discussed, the contribution of stromal cells to the tumor cell gene expression profile seems small [34]. A possible increase in stromal content cannot provide an explanation for this result. The effect at the protein level should also be assessed. Furthermore an alternative might be experimental bias due to the small sample size of node positive rectal cancer cases.

### 8q gain and BOP1

Gain of chromosome 8q and a higher expression of *BOP1 *were both observed in most carcinomas (Figure 2). BOP1 is a member of the Pes1-Bop1 complex, involved in ribosome biogenesis [35]. Killian *et al. *proposed that *BOP1 *deregulation leads to altered chromosome segmentation and chromosomal instability in colorectal cancer [29]. They showed that *BOP1 *copy number increase was associated with *BOP1 *gene over-expression, in concordance with our results. *BOP1 *is located on 8q24, close to the *MYC *oncogene. However, gene dosage increase of *BOP1 *was independent from that of *MYC *and was more frequent than *MYC *over-expression, suggesting that *BOP1 *over-expression may be one of the main oncogenic consequences of 8q24 amplification in colorectal cancer. In our data series, gain of 8q, and consequently *BOP1 *over expression, was predominantly observed in cases with high chromosomal instability.

**Figure 5:**
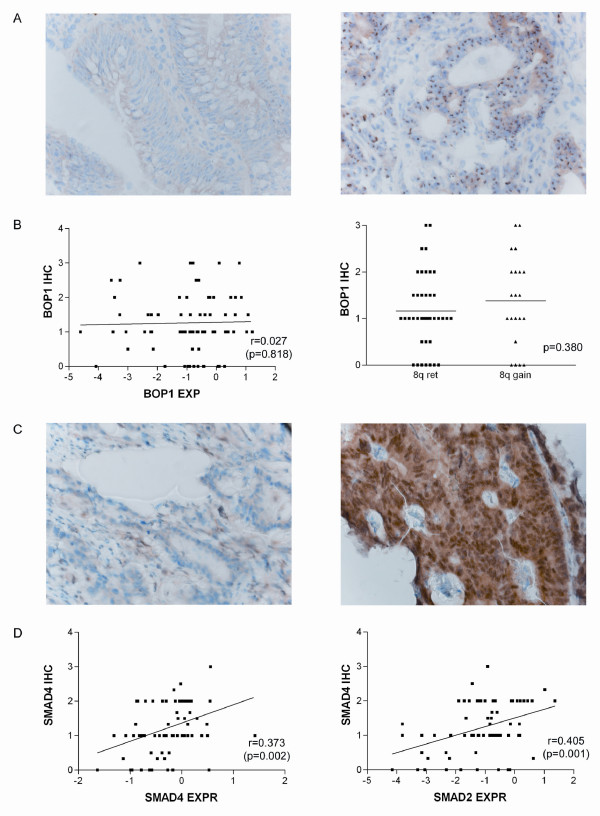
**BOP1 and SMAD4 immunohistochemical staining**. A. Example of BOP1 immunohistochemical staining in two carcinomas; the left picture shows a weak expression (IHC score 1), the right picture, a very strong expression (score 3). B. Correlation plot of *BOP1 *mRNA expression (x-axis) and BOP1 protein expression (y-axis) (left) and BOP1 immunohistochemical staining (y-axis) related to 8q gain(x-axis) (right). C. Example of SMAD4 immunohistochemical staining in two carcinomas; the left picture shows loss of SMAD4 expression (score 1), the right picture positive expression (score 3). D. Correlation plot of *SMAD4 *gene expression (x-axis) and SMAD 4 protein expression (y-axis) (left) and correlation plot of *SMAD2 *gene expression (x-axis) and SMAD 4 protein expression (right) (y-axis).

BOP1 protein expression was measured through immunohistochemistry on rectal tumor tissue slides. Specific cases with high *BOP1 *mRNA expression showed very intense nucleolar BOP1 staining (Figure 5A), but a direct correlation between both parameters was not established (Figure 5B). Comparing the mean BOP1 protein expression between samples with 8q retention and 8q gain revealed a slight, although not significant, increase in the samples with gain (1.38 vs 1.16 relative protein expression) (Figure 5B). Post-transcriptional and post-translational mechanisms are likely to influence protein expression, possibly blurring the correlation between mRNA and protein levels for this gene. In such a case, gene expression data and immunohistochemistry results must be considered independently because each can provide clinically meaningful information [36]. Alternatively, the difference in gene expression might be too subtle to detect with immunohistochemistry.

### 18q loss and SMAD2

*SMAD2, SMAD4 *and *DCC *are indicated as the prominent tumor suppressor genes on 18q [30,37]. We found that *SMAD2 *was significantly less expressed in the cases with 18q loss (Figure 2), and *SMAD2 *and *SMAD4 *were both down-regulated in the more advanced tumor stages (Limma-analysis, data not shown). SMAD proteins mediate TGF-β signaling to regulate cell growth and differentiation [38]. LOH in combination with *SMAD4 *mutations is a well studied phenomenon in CRC, and *SMAD4 *gene mutations are related to advanced tumor stage [39,40]. Immunohistochemistry was technically not feasible for SMAD2 but was feasible for SMAD4, which is also located on chromosome18q21.1. Therefore, we tested whether SMAD4 protein expression was correlated to *SMAD4 *and *SMAD2 *gene expression, which was indeed the case (r = 0.373 (p = 0.002) and r = 0.405, (p = 0.001)) (Figure 5C, D).

According to Knudson's "two hit hypothesis", both copies of a tumor suppressor gene should be deleted by a mutation or allelic loss to reduce protein dosage [41]. In our study, half of the cases showed physical loss of 18q and thus deletion of one of the SMAD2 alleles. An additional hit such as a mutation can then be expected, leading to the observed reduction in protein expression. However, mutation analysis for *SMAD2 *did not reveal any mutations in this sample series (data not shown). In the literature, mutation rates vary between 0 and 30% for *SMAD2 *and *SMAD4 *[37,42]. Recently, Alberici et al. showed haploinsufficiency for the *SMAD4 *locus in mouse models for colorectal cancer, giving an explanation for the relatively low mutation rate observed [43]. Consequently, the loss of one allele already leads to reduced SMAD4 protein expression and altered TGF-β signaling. The same principle might apply to SMAD2 and explain our findings, where only one copy of 18q is lost and no mutation is found in the *SMAD2 *gene, but reduced gene expression is observed.

### Genes on chromosome 20q

Genes on chromosome 20 showed the highest fold change in expression in comparison with genes on the other chromosomes (Additional file 1). Two interesting genes were in the top five overexpressed genes: *STMN3 *and *TGIF2*. *STMN3 *is overexpressed in various human malignancies and plays a role in regulation of the cell cycle [28,44]. In oral squamous-cell carcinoma, the overexpression of *STMN3 *was correlated with tumor progression and poor prognosis. Kouzu *et al. *emphasized the potential role of *STMN3 *as a biomarker and therapeutic target for oral squamous-cell carcinoma [28]. TGIF2 was shown to interact with TGF-β-activated SMADS and repress TGF-β responsive transcription [45]. Limma-analysis revealed that TGIF2 was among the ten most significant genes in the adenoma-carcinoma comparison. In ovarian cancer cell lines, amplification of 20q correlated strongly with *TGIF2 *over-expression [46]. A recent study subtracted a chromosomal instability gene expression profile from 12 different cancer data sets. This 25 gene set contained, among others, *TGIF2*, indicating that this gene plays a role in chromosomal instability [47].

## Conclusion

Several studies have integrated global patterns of gene expression and genomic data in colorectal cancer, with divergent results. While some studies reported a direct correlation between gene expression and chromosomal aberrations [16,17,19,48], others reported that amplification does not necessarily lead to expression up-regulation[20]. Similarly, genomic regions with deletions show reduced expression while amplified regions exhibit heterogeneous expression [18]. We first determined whether copy number alterations have an effect on gene expression and saw that changes in genomic regions and gene expression are usually in the same direction. We then performed supervised analysis to find target genes on the affected chromosomes. We identified one well known CRC gene (*SMAD2*) and several other genes (*EFNA1, BOP1, TGIF2 *and *STMN3*) that possibly could be used for rectal cancer characterization. It will be interesting to determine if these molecules can discriminate benign adenomas from adenomas containing an invasive focus and carcinomas with lymph node metastasis. To validate the impact of these genes in rectal carcinogenesis it will be worthwhile to study them for mRNA expression and at the immunohistochemical level in representative cases with normal, adenoma and carcinoma tissue from single patients. Unfortunately frozen material of all those tumor stadia was not available for the subjects of this study. Studies in other cancer types have successfully applied supervised methods [49,50]. Garraway et al. performed supervised analysis on cell lines with and without 3p amplifications and identified MITF as a new melanoma oncogene.

In conclusion, gene expression values in regions with genomic changes are altered in the same direction. We analyzed gene expression data in relation to specific chromosomal aberrations involved in the progression from adenoma to carcinoma. By not focusing directly on tumor stages, but rather on genomic aberrations related to tumor stages, we identified several specific genes on altered chromosomes in rectal cancer. Specific genes, identified by such integration methods, could be of additional value to further explain rectal tumorigenesis.

## Competing interests

The authors declare that they have no competing interests.

## Authors' contributions

EL performed the experiments, data analysis and preparation of the manuscript. RE and NM assisted in the experiments. JO assisted in data analysis. EG, TK, CV provided the tumor material. PE, RT, TW, HM initiated the study and supervised the data generation and analyses. All authors read and approved the final manuscript.

## Pre-publication history

The pre-publication history for this paper can be accessed here:



## Supplementary Material

Additional file 1 **Supplementary Tables. **Table 1 contains the data of the Limma analysis for comparison of the different tumor groups (a) and the "progression" analysis (b). Supplementary Table 2 contains p-values for genes of the progression analysis on chromosome comparisons. Supplementary Table 3 contains the differentially expressed genes for the six genomic regions of interest. Click here for file

Additional file 2 **Figure. **Contains graphs of analysis. Click here for file
