# Supplementary material for: Integrating chromosomal aberrations and gene expression profiles to dissect rectal tumorigenesis
Source: BMC Cancer. 2008 Oct 29;8:314. doi: 10.1186/1471-2407-8-314 (PMC2584339; doi:10.1186/1471-2407-8-314)
Supplement: Additional file 2 — Figure. Contains graphs of analysis. [file 1471-2407-8-314-S2.ppt]

## Slide 1
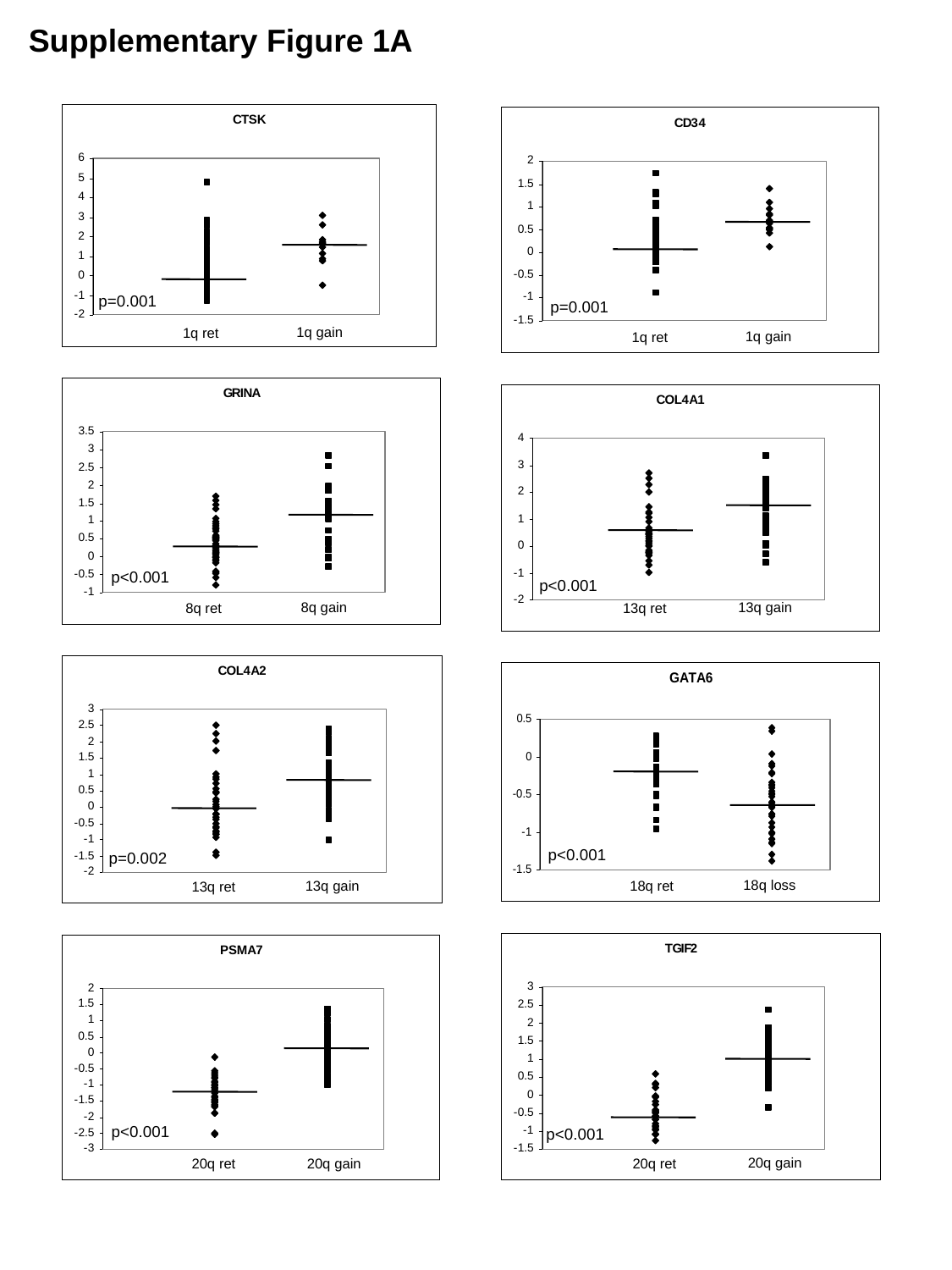

Supplementary Figure 1A
p=0.001
1q gain
 1q ret
p=0.001
1q gain
 1q ret
p<0.001
8q gain
 8q ret
p<0.001
13q gain
 13q ret
p=0.002
13q gain
 13q ret
p<0.001
18q loss
 18q ret
p<0.001
20q gain
 20q ret
p<0.001
20q gain
 20q ret

## Slide 2
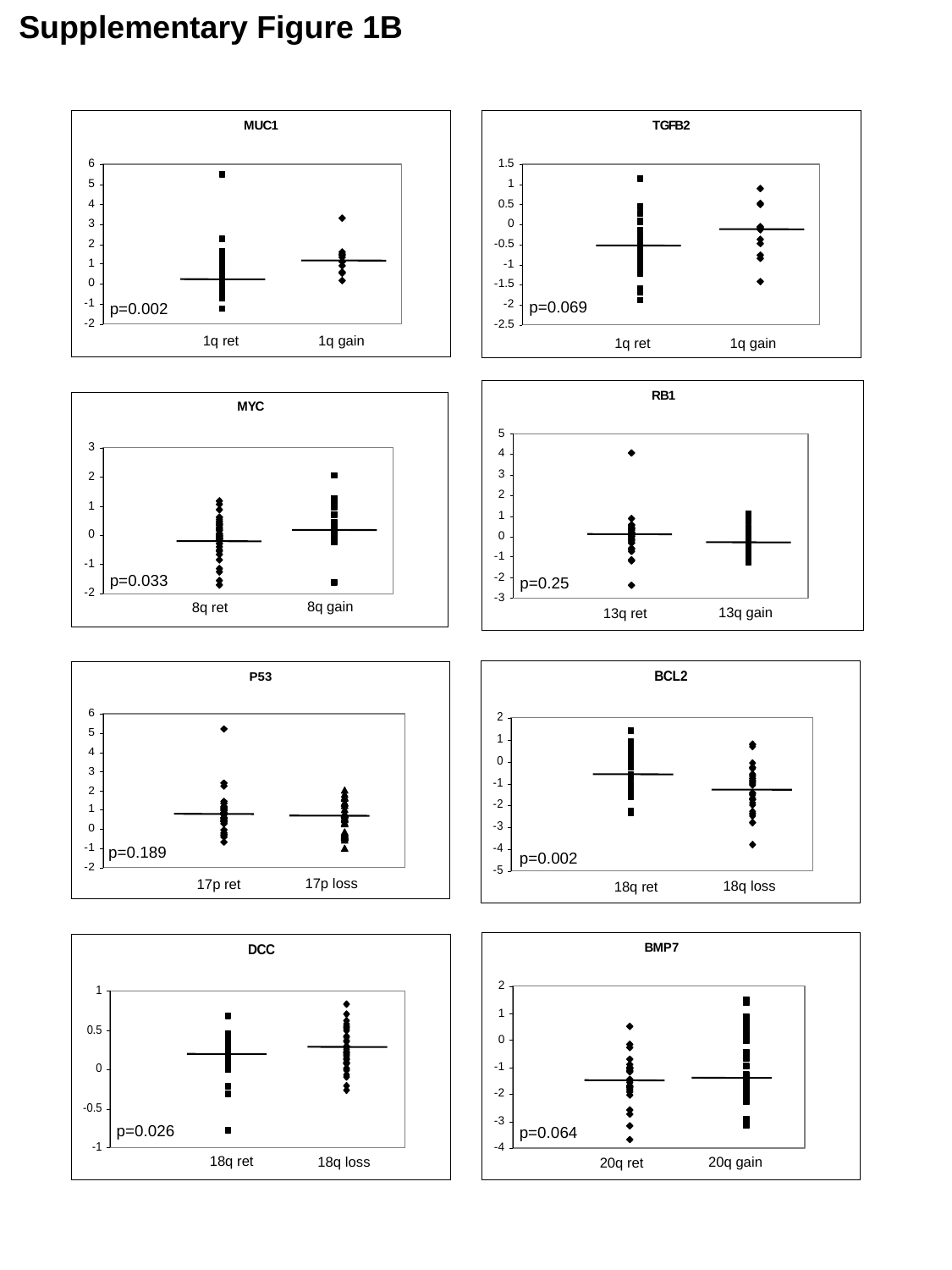

Supplementary Figure 1B
p=0.002
1q gain
 1q ret
p=0.069
1q gain
 1q ret
p=0.25
13q gain
 13q ret
p=0.033
8q gain
 8q ret
p=0.002
18q loss
 18q ret
p=0.189
17p loss
 17p ret
p=0.064
20q gain
 20q ret
p=0.026
 18q ret
18q loss
